# Assessment of lung deformation in patients with idiopathic pulmonary fibrosis with elastic registration technique on pulmonary three-dimensional ultrashort echo time MRI

**DOI:** 10.1186/s13244-023-01555-x

**Published:** 2024-01-23

**Authors:** Xiaoyan Yang, Pengxin Yu, Haishuang Sun, Mei Deng, Anqi Liu, Chen Li, Wenyan Meng, Wenxiu Xu, Bingbing Xie, Jing Geng, Yanhong Ren, Rongguo Zhang, Min Liu, Huaping Dai

**Affiliations:** 1 Department of Pulmonary and Critical Care Medicine, General Hospital of Ningxia Medical University, Yinchuan, 750004 Ningxia China; 2 National Center for Respiratory Medicine, National Clinical Research Center for Respiratory Diseases, Institute of Respiratory Medicine, Chinese Academy of Medical Sciences, Department of Pulmonary and Critical Care Medicine, Center of Respiratory Medicine, China-Japan Friendship Hospital, 2 Yinghua Dong Street, Hepingli, Chao Yang District, Beijing, 100029 China; 3 Institute of Advanced Research, Infervision Medical Technology Co., Ltd, Beijing, 100025 China; 4 Department of Radiology, China-Japan Friendship Hospital, 2 Yinghua Dong Street, Hepingli, Chao Yang District, Beijing, 100029 China

**Keywords:** Elastic registration, Idiopathic pulmonary fibrosis, Magnetic resonance imaging, Ultrashort echo time

## Abstract

**Objective:**

To assess lung deformation in patients with idiopathic pulmonary fibrosis (IPF) using with elastic registration algorithm applied to three-dimensional ultrashort echo time (3D-UTE) MRI and analyze relationship of lung deformation with the severity of IPF.

**Methods:**

Seventy-six patients with IPF (mean age: 62 ± 6 years) and 62 age- and gender-matched healthy controls (mean age: 58 ± 4 years) were prospectively enrolled. End-inspiration and end-expiration images acquired with a single breath-hold 3D-UTE sequence were registered using elastic registration algorithm. Jacobian determinants were calculated from deformation fields and represented on color maps. Jac-mean (absolute value of the log means of Jacobian determinants) and the Dice similarity coefficient (Dice) were compared between different groups.

**Results:**

Compared with healthy controls, the Jac-mean of IPF patients significantly decreased (0.21 ± 0.08 vs. 0.27 ± 0. 07, *p* < 0.001). Furthermore, the Jac-mean and Dice correlated with the metrics of pulmonary function tests and the composite physiological index. The lung deformation in IPF patients with dyspnea Medical Research Council (MRC) ≥ 3 (Jac-mean: 0.16 ± 0.03; Dice: 0.06 ± 0.02) was significantly lower than MRC1 (Jac-mean: 0. 25 ± 0.03, *p* < 0.001; Dice: 0.10 ± 0.01, *p* < 0.001) and MRC 2 (Jac-mean: 0.22 ± 0.11, *p* = 0.001; Dice: 0.08 ± 0.03, *p* = 0.006). Meanwhile, Jac-mean and Dice correlated with health-related quality of life, 6 min-walk distance, and the extent of pulmonary fibrosis. Jac-mean correlated with pulmonary vascular-related indexes on high-resolution CT.

**Conclusion:**

The decreased lung deformation in IPF patients correlated with the clinical severity of IPF patients. Elastic registration of inspiratory-to-expiratory 3D UTE MRI may be a new morphological and functional marker for non-radiation and noninvasive evaluation of IPF.

**Critical relevance statement:**

This prospective study demonstrated that lung deformation decreased in idiopathic pulmonary fibrosis (IPF) patients and correlated with the severity of IPF. Elastic registration of inspiratory-to-expiratory three-dimensional ultrashort echo time (3D UTE) MRI may be a new morphological and functional marker for non-radiation and noninvasive evaluation of IPF.

**Key points:**

• Elastic registration of inspiratory-to-expiratory three-dimensional ultrashort echo time (3D UTE) MRI could evaluate lung deformation.

• Lung deformation significantly decreased in idiopathic pulmonary fibrosis (IPF) patients, compared with the healthy controls.

• Reduced lung deformation of IPF patients correlated with worsened pulmonary function and the composite physiological index (CPI).

**Graphical Abstract:**

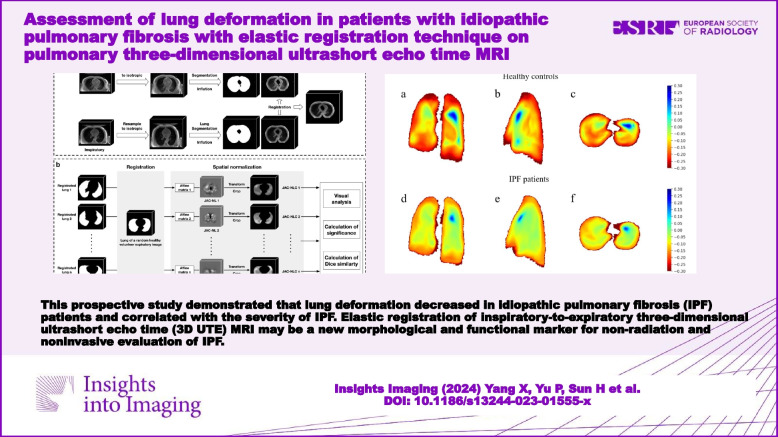

**Supplementary Information:**

The online version contains supplementary material available at 10.1186/s13244-023-01555-x.

## Introduction

Idiopathic pulmonary fibrosis (IPF) is a chronic, progressive fibrotic lung disease, ultimately causing to respiratory failure and mortality [1]. At present, pulmonary function tests (PFTs) and high-resolution computed tomography (HRCT) are the most important methods for the diagnosis and evaluation of IPF [2, 3]. However, PFTs only offer a general overview of lung function and do not provide detailed information about the regional function of IPF, while visual assessments of HRCT images are subjective and depend on the expertise of the radiologist, which can limit their reliability and reproducibility.

The elastic registration algorithm is a specific type of imaging registration method designed to determine the transformation necessary to align a source image with a target image. This is particularly useful for images taken at different time points or stages of the same time, as the algorithm can automate and quantify the differences between the two images [4, 5]. Recently, the elastic registration algorithm has been paid attention to assessing lung deformation in acute lung injury [6], chronic obstructive pulmonary disease [7, 8], and asthmatic [9]. Chassagnon et al. used elastic registration on HRCT images to evaluate the morphological and functional deterioration of systemic sclerosis-related interstitial lung disease (SSc-ILD) [10]. Moreover, it has been shown that performing elastic registration between baseline and follow-up HRCT can be advantageous in quantitatively assessing the morphological deterioration of lung shrinkage that is characteristic of IPF [11].

To minimize the cumulative radiation exposure associated with frequent CT scans, ultrashort echo time (UTE) and zero echo time (ZTE) sequences have been introduced into clinical MRI protocols. These sequences can depict structural and functional alterations and can even be used in children [12, 13]. UTE is capable of generating CT-like contrast in the lung parenchyma [14], and 3D-UTE MRI has the high reproducibility to identify the imaging features of IPF and evaluate the extent of pulmonary fibrosis [15].

Using elastic registration analysis of inspiratory-to-expiratory UTE MRI, Chassagnon et al. demonstrated a reduction in lung base respiratory deformation in patients with systemic sclerosis-related pulmonary fibrosis, compared to those without fibrosis [16]. Thus, we hypothesized that lung deformation with elastic registration technique on 3D-UTE MRI could be used as a potential biomarker for the severity of IPF. We aimed to analyze the correlation of lung deformation with pulmonary function, dyspnea, exercise tolerance, health-related quality of life (HRQoL), and the extent of pulmonary fibrosis on HRCT in patients with IPF.

## Materials and methods

### Study design and cohort

**Fig. 1:**
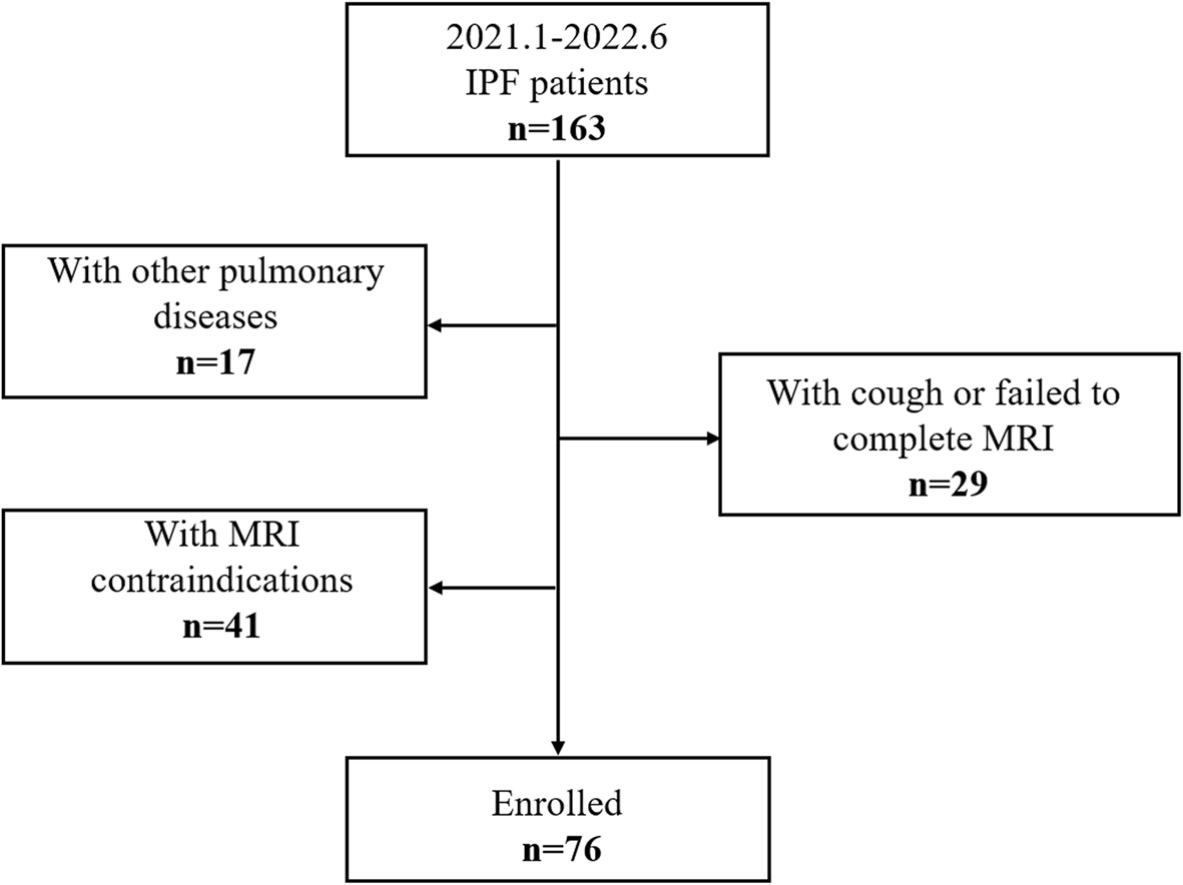
The flowchart of inclusion and exclusion criteria of idiopathic pulmonary fibrosis patients

This prospective single-center cohort study was approved Institutional review board of our hospital (2019-123-K85-1). All participants provided written informed consent. From January 2021 and June 2022, patients with IPF and age- and gender-matched healthy controls were included. IPF patients were diagnosed by the multidisciplinary team using the diagnostic criteria established by the American Thoracic Society, European Respiratory Society, Japanese Respiratory Society, and Latin American Thoracic Association (ATS/ERS/JRS/ALAT) in 2018 [1]. The people with normal HRCT findings and PFTs were included in the control group. Exclusion criteria include (I) participants with cancer or infection; (II) participants with MRI contraindications, such as a pacemaker or claustrophobia; and (III) participants who failed to complete MRI scan or had poor image quality with the obvious artifact. All participants underwent MRI, HRCT, and PFTs within 24 h. Figure 1 showed the flowchart of how participants were enrolled.

The severity of resting dyspnea in IPF patients was assessed using the Medical Research Council (MRC) scale [17]. Health-related quality of life was evaluated based on a respiratory-specific questionnaire, the St. George’s Respiratory Questionnaire (SGRQ) [18]. This questionnaire encompasses three domains, including respiratory symptoms, activity, and the psychosocial impact of the disease, and scores ranging from 0 to 100 indicate a worse quality of life. Additionally, the study also measured the 6-min walking distance (6-MWD) of all patients.

### Pulmonary function tests

All participants underwent PFTs (MasterScreen, Vyaire Medical GmbH) according to the standards of ATS/ERS [19]. PFT measurements included the percentage of predicted forced vital capacity (FVC%), percentage of forced expiratory volume in 1 s (FEV1%), FEV1/FVC%, percentage of predicted total lung capacity (TLC%), and percentage of predicted DLco corrected for measured hemoglobin (DLco%). Additionally, the composite physiological index (CPI) was calculated using the following formula: CPI = 91 − (0.65 × %predicted DLCO) − (0.53 × %predicted FVC) + (0.34 × %predicted FEV1) [20].

### HRCT and quantitative analysis

All participants underwent HRCT using multidetector CT systems (Toshiba Aquilion ONE TSX-301C/320; Philips iCT/256) in full inspiration. The chest was scanned from the lung apex to the lowest hemidiaphragm in a single breath-hold in a supine position with the following acquisition parameters and reconstruction parameters: tube voltage = 100–120 kV, tube current = 100–300 mAs, section thickness = 0.625–1 mm, table speed = 39.37 mm/s, gantry rotation time = 0.8 s, and reconstruction increment = 1–1.25 mm.

Segmentation of lung and fibrosis lesions was performed in the software InferScholar (https://www.infervision.com/) [21]. The full lung region was automatically segmented and then manually corrected by one radiologist. According to Christe et al. [22], ground-glass opacities (GGO), reticulation, and honeycombing signs on HRCT were outlined (Supplementary figure 1). The extent of lung fibrosis was quantified as the percentage of the volume occupied by reticulation and honeycombing in relation to the total lung volume.

According to previous studies [23, 24], pulmonary vessels on HRCT were automatically segmented using an integrated segmentation method that employs automated algorithms (the FACT-Digital Lung Workstation, Dexin) and subsequently reviewed by the radiologist and manually corrected (Supplementary figure 2). The main vascular parameters quantified included the volume, number, and tortuosity of pulmonary vessel branches of total pulmonary vascular (TPV), pulmonary vein vascular (PVV), and pulmonary artery vascular (PAV).

### Magnetic resonance imaging

All patients underwent chest MRI using a 1.5T MRI scanner (MAGNETOM Aera; Siemens Healthcare) with an 18-channel phased-array body coil. Two three-dimensional, ultrashort echo time, gradient echo spiral volume interpolated breath-hold examination sequences were acquired during a single respiratory phase, one following a full inspiration and the other after a full expiration. Both sequences were performed in the coronal plane and had a duration of 15 s. The key parameters as following: repetition time = 2.73 msec; echo time = 0.05 msec; flip angle = 5°; field-of-view = 500 × 500 mm; slice thickness = 2.5 mm; matrix = 240 × 240; in-plane resolution = 2.08 × 2.08 mm; spiral duration = 1800 μsec. Image reconstructions used the non-uniform Fourier transform (NUFT) method. To promise full inspiration and expiration, each participant was trained to practice deep breathing in the supine position before the MRI scan.

### Elastic registration

**Fig. 2:**
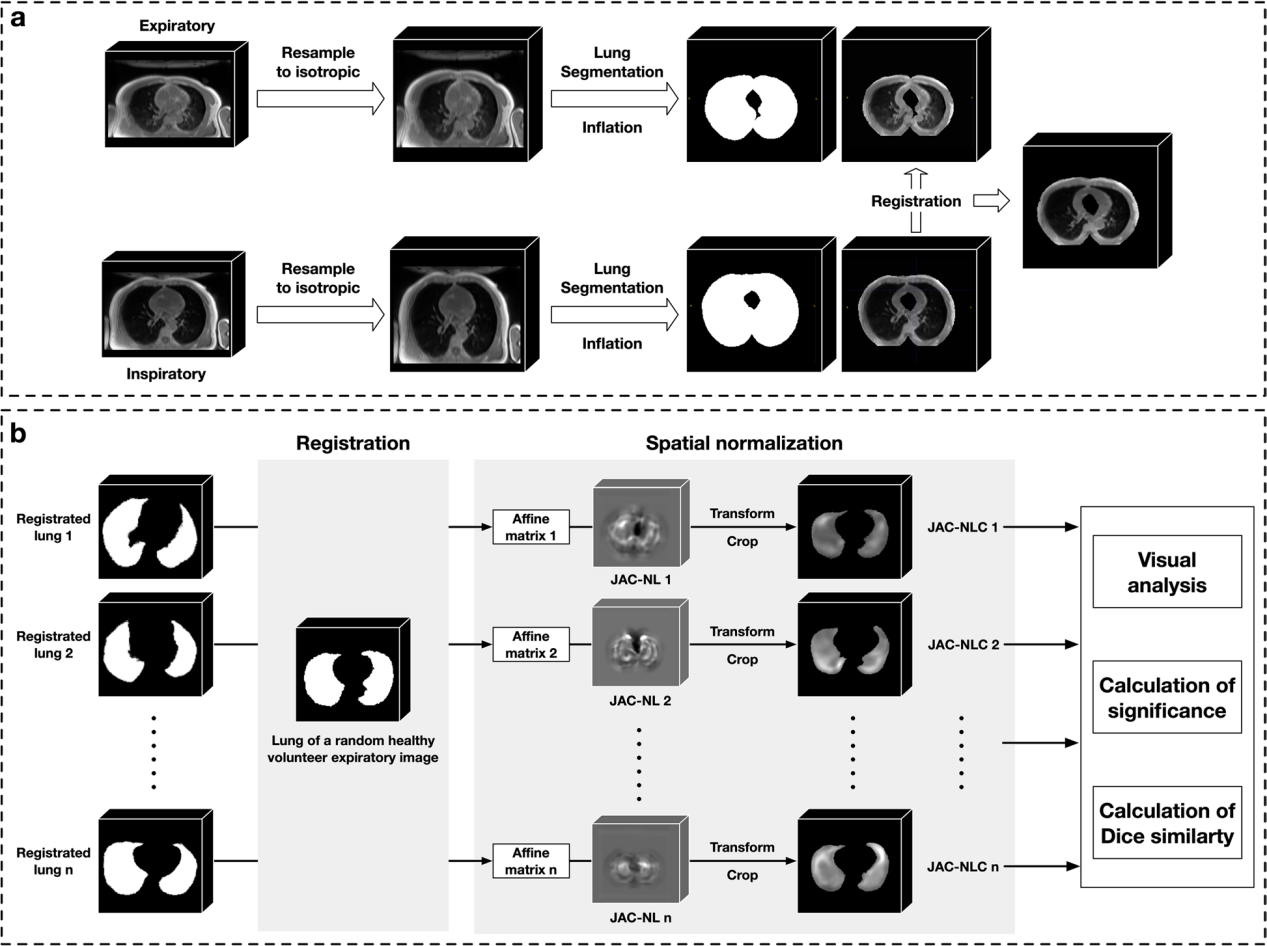
The flowchart of elastic registration. **a** The procedure of inspiratory-to-expiratory imaging registration of transversal lung MRI. The inspiratory and expiratory images of the patient were subjected to isotropic resampling and then the lung and peri-lung regions were extracted as regions of interest (ROI). Based on the ROI only, the inspiratory image is registered to the expiratory image to obtain the registered inspiratory image. **b** The steps of Jacobian determinant analysis. First, a common space is defined, and the expiratory images of all participants are registered to this common space, and the registration affine matrix of each participant is obtained. Then, based on each participant's affine matrix, a quadratic transformation of the respective Jacobian determinant (JAC) is performed. Finally, participants were grouped, and various comparisons and analyses were made between groups based on the registered JAC

Figure 2a shows the elastic registration of inspiratory and expiratory UTE images. First, all inspiratory and expiratory UTE images were preprocessed by isotropic sampling with 1 mm. Then, the lung regions were automatically segmented with the InferScholar (https://www.infervision.com/). An experienced observer checked lung segmentation results to ensure that the lungs were completely and accurately identified. The fined lung segmentation was then dilated by ten pixels to contain the peri-lungs. ElasticSyN algorithm was used to perform elastic registration of inspiratory (moving) to expiratory (fixed) images. This algorithm is integrated into a stable and powerful open-source software called Advanced Normalization Tools (ANTs) (https://github.com/ANTsX/ANTs) [25].

Two metrics are used to evaluate the quality of elastic registration. The first metric is the intersection over union (IoU) [26], which measures the overlap degree between the lung area of the inspiratory image after registration and that of the expiratory image. The second metric measured the distance between the landmark in the inspiratory image after registration and that in the expiratory image. Specifically, an observer manually places two categories of landmarks A and B on both inspiratory and expiratory images (Supplementary figure 3). After the registration is completed, the landmarks on the inspiratory image are automatically registered to the expiratory image. The landmarks coordinates were collected to calculate the mean distances of two landmarks: DA, the distance between registered landmark-A and original landmark-A in the expiratory image; and DB, the distance between two landmark-Bs. To verify the repeatability and stability of the registration method, two times of respiratory phases (inspiratory and expiratory) were acquired for each participant. IoU, DA, and DB are calculated for each respiratory phase, and the consistency between the two phases is calculated.

### Analysis of lung deformation

For each participant, the Jacobian determinant (JAC) was calculated through the deformation field resulting from respiratory registration. JAC is a matrix of the same size as the expiratory image, where each value indicates whether the corresponding voxel stretched (greater than 1) or shrunk (less than 1). The JAC of each participant was first normalized by the ratio of their inspiratory and expiratory lung volumes, resulting in JAC-N. To better distinguish stretched and shrunk numerically, JAC-N is performed logarithmically to obtain JAC-NL, where a positive value implies voxel stretch, a negative value implies voxel shrinkage, and 0 implies no change.

Figure 2b shows analysis of lung deformation. A full-expiratory image of a healthy volunteer was randomly selected as a common space. The JAC-NL of each participant was registered to this common space based on their lung mask, resulting in JAC-NLC.

For visual analysis, JAC-NLC of all IPF patients and healthy volunteers were averaged to obtain the corresponding average Jacobian determinant respectively: IAJ for IPF patients and HAJ for healthy volunteers. Then, project the IAJ and HAJ to different directions along the *x*, *y*, and *z* axis to get the visual deformation on coronal, sagittal, and axial views.

For quantitative analysis, Jac-mean, defined as the absolute value of mean JAC-NLC for each participant, was calculated for inter-group comparative analysis. In addition, followed Chassagnon et al. [16], we defined marked deformation lung areas as those with JAC-NLC values below 0.15 (cutoff value). First, healthy deformation template is segmented by using the cutoff value on the HAJ, which represents the lung motion pattern when breathing in a healthy volunteer group. Then, Dice similarity coefficient was used to calculate the consistency of the marked deformation area with healthy deformation template for each IPF patient. Specifically, the area that overlaps the marked deformation area of each IPF patient and the HAJ is recorded as true positive (TP), the marked deformation area that belongs to the HAJ but not the IPF patient is recorded as false negative (FN), and the marked deformation area belonging to the IPF patient but not belonging to the HAJ is recorded as false positive (FP), then the Dice similarity coefficient is calculated as 2 × TP/(2 × TP + FN + FP). For each IPF patient, the larger the Dice, the more consistent the lung motion pattern is with that of healthy volunteer group, and, conversely, the lower the consistency.

All the aforementioned procedures were implemented using the Python programming language (version 3.8; Python Software Foundation) within the Ubuntu operating system environment (version 16.04; Canonical Ltd.).

### Statistical analysis

All statistical analyses were performed with SPSS 26.0 (IBM Corp, Armonk). The unpaired *t*-test and Mann-Whitney *U* test (quantitative data) or chi-square test (categorical variables) were used for comparing different groups. The Spearman correlation coefficient was used to analyze the correlation between the lung deformation and severity of IPF. The Bland-Altman analysis [27] and intraclass coefficients (ICC) were used to determine the repeatability of the method. ICCs were classified from null (= 0) to very good (> 0.80) and almost perfect (> 0.95) [28]. Statistical significance was assumed when two-tail *p* ≤ 0.05.

## Results

### Baseline characteristics

Seventy-six patients with IPF (72 men, mean age: 62 ± 6 years) and 62 healthy controls (58 men, mean age: 58 ± 4 years) were prospectively enrolled. Table 1 showed the demographic characteristics of IPF patients and healthy controls. Gender and body mass index (BMI) were comparable between two groups. The mean values of FVC% predicted (80.9 ± 14.5), FEV1% (82.4 ± 14.4), TLC% (67.7 ± 11.1), and DLco% (54.8 ± 15.2) predicted for IPF patients significantly decreased in comparison with the controls. CPI in IPF patients (39.1, [IQR: 32.4, 48.1]) was significantly more than healthy controls (5.6, [IQR: − 8.0, 13.7], *p* < 0.001).

**Table 1:** The characteristics of all participants

Patients	IPF (*N* = 76)	Control (*N* = 62)	*t/χ*^*2*^	*p*
Mean age (years old)	62 ± 6	58 ± 4	1.957	0.055
Gender, male, *n* (%)	72 (94.7%)	58 (93.5 %)	0.088	0.767
Height (cm)	168.3 ± 7.1	169.8 ± 5.8	− 1.318	0. 190
Weight (kg)	72.1 ± 11.2	71.7 ± 7.4	0.203	0.839
BMI (m/kg^2^)	25.0 ± 2.7	24.9 ± 2.3	0.294	0.769
Pulmonary function				
FVC % predicted	80.9 ± 14.5	105.9 ± 14.1	− 10.229	< 0.001^*^
FEV1% predicted	82.4 ± 14.4	100.3 ± 12.2	− 7.775	< 0.001^*^
FEV1/FVC% predicted	81.4 ± 6.1	81.7 ± 8.8	− 0.236	0.814
TLC % predicted	67.7 ± 11.1	99.2 ± 13.9	− 14.753	< 0.001^*^
DLCO % predicted	54.8 ± 15.2	102.3 ± 18.1	− 16.801	< 0.001^*^
CPI	39.1 (32.4, 48.1)	5.6 (− 8.0, 13.7)	− 9.845	< 0.001^*^
6-MWD (m)	462.7 ± 67.1	\		
Resting dyspnea (MRC), *n* (%)				
0	0	\		
1	24 (31.6%)	\		
2	34 (44.7%)	\		
3	12 (15.8%)	\		
4	6 (7.9%)	\		
HRQoL				
Respiratory symptoms (%)	51.5 (32.1, 63.5)	\		
Activity (%)	42.1 (27.6, 55.6)	\		
Psychosocial impact (%)	16.9 (7.8, 28.4)	\		
Total (%)	31.1 (14.9, 40.4)	\		
The percentage of pulmonary fibrosis on HRCT				
Pulmonary fibrosis (%)	8.3 (4.8, 16.3)	\		
Pulmonary vascular-related indexes				
TPV volume (ml)	181.22 (160.61, 219.29)	194.56 (182.75, 211.33)	− 1.627	0.104
TPV number	1706 (1422, 2185)	2716 (2414, 2998)	− 8.408	< 0.001^*^
TPV tortuosity	1.12 (1.09, 1.18)	1.07 (1.05, 1.08)	− 8.396	< 0.001^*^
PVV volume (ml)	94.55 (80.44, 114.72)	95.78 (86.74, 99.98)	− 0.634	0.526
PVV number	930 (722, 1139)	1320 (1190, 1478)	− 6.353	< 0.001^*^
PVV tortuosity	1.11 (1.08, 1.16)	1.07 (1.05, 1.08)	− 7.087	< 0.001^*^
PAV volume (ml)	90.20 (78.32, 105.80)	99.37 (92.05, 113.25)	− 2.877	0.004
PAV number	805 (550, 956)	1352 (1173, 1476)	− 9.487	< 0.001^*^
PAV tortuosity	1.12 (1.10, 1.12)	1.06 (1.05, 1.08)	− 8.194	< 0.001^*^

^*^p < 0.05, *IPF*, idiopathic pulmonary fibrosis; *BMI*, body mass index; *FEV1*, forced expiratory volume; *FVC*, forced vital capacity; *TLC*, total lung capacity; *DLco*, diffusing capacity of the lungs for carbon monoxide; *CPI*, composite physiological index; *6-MWD*, 6 min-walk distance; *HRQoL*, health-related quality of life; *ILD*, interstitial lung disease; *HRCT*, high-resolution computed tomography; *TPV*, total pulmonary vascular; *PVV*, pulmonary vein vascular; *PAV*, pulmonary artery vascular

### Repeatability of elastic registration algorithm

**Fig. 3:**
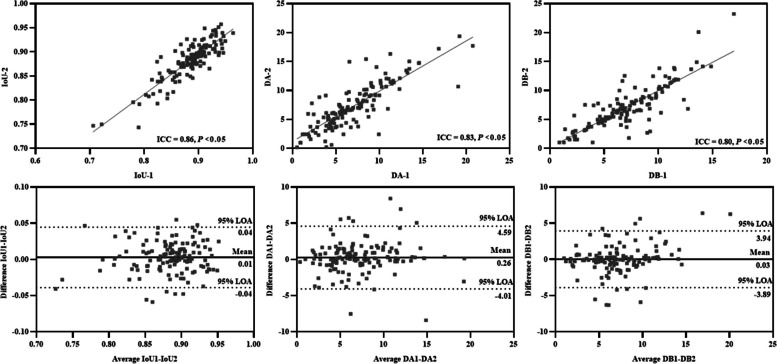
The inter-observer agreement (Intraclass coefficients and Bland-Altman plot agreement) of intersection over union (IoU), distance A (DA), and distance B (DB) between the two respiratory phases. 95% LOA, 95%limits of agreement

The mean value of IoU was 0.88 ± 0.03. In addition, the mean distance between the landmark as (DA) and landmark-Bs (DB) were 7.1 ± 3.8 mm and 7.0 ± 3.1 mm, respectively. The inter-observer agreement between the two respiratory phases was very good (IoU, ICC = 0.86; distance A, ICC = 0.83; distance B, ICC = 0.80) (Fig. 3). The mean difference and 95% limits of agreement (95% LOA) between two respiratory phrases for IoU, DA, and DB were 0.01 (− 0.04 to 0.04), 0.26 (− 4.01 to 4.59), and 0.03 (− 3.89 to 3.94), respectively.

### Comparison of lung deformation between IPF patients and the controls

**Fig. 4:**
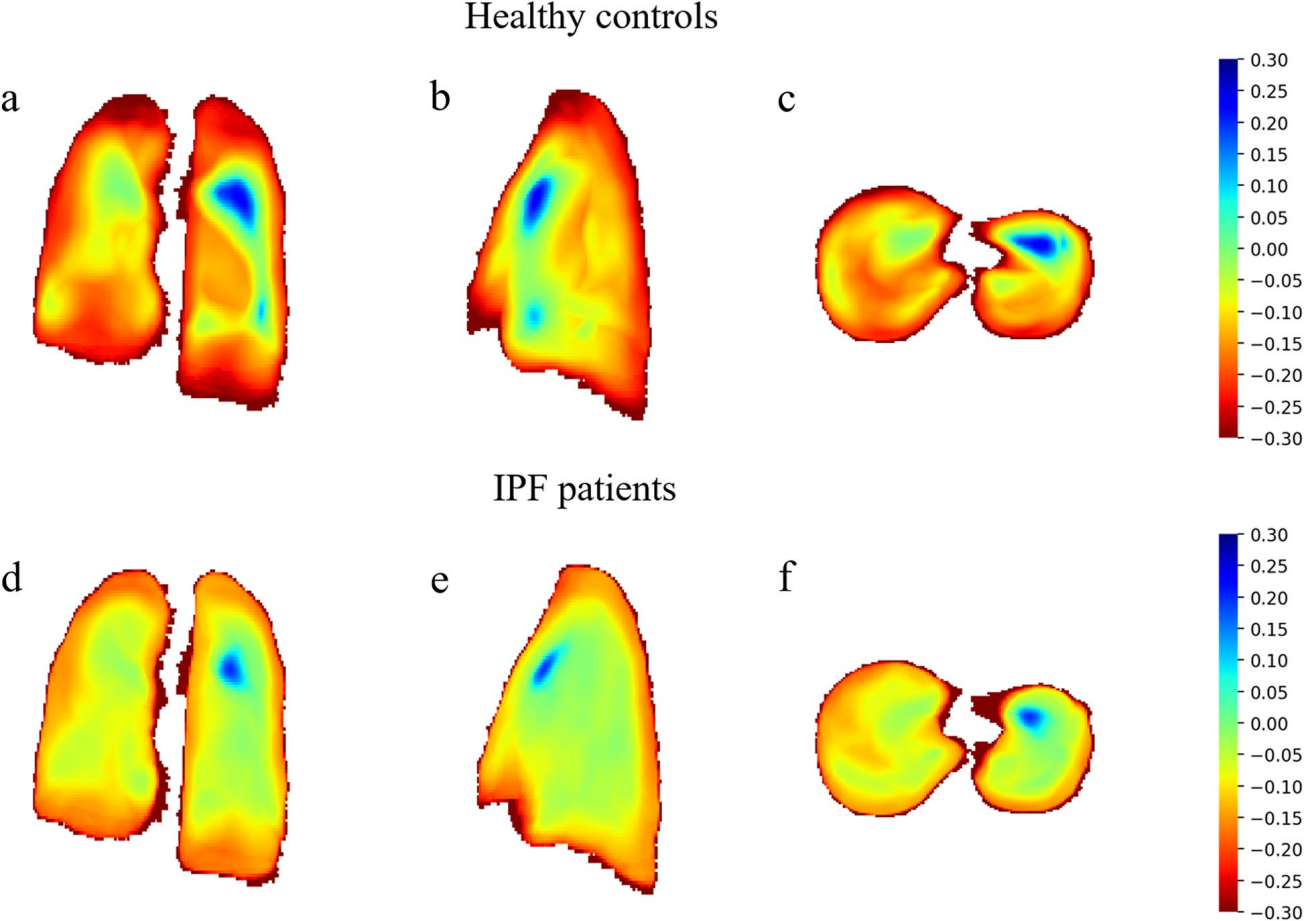
The coronal, sagittal, and transversal projection of mean of Jacobian determinants in IPF patient and control. Marked shrinkage areas (in red) are found in the peripheral region of lung bases in healthy volunteers (**a**–**c**) and study participants with idiopathic pulmonary fibrosis (IPF) (**d**–**f**) in MRI. In corresponding areas, the lungs of IPF patients show little deformation with mean Jacobian determinant

Figure 4 displays color maps illustrating the differences in lung deformation between IPF patients and healthy controls. These deformation maps were generated in the coronal, sagittal, and transversal directions. Comparing the deformation patterns between the two groups, healthy controls had a significant deformation during expiration, particularly in the peripheral regions of the lungs. In contrast, IPF patients exhibited the decreased deformation in the peripheral regions of the lungs during expiration. The Jac-mean of IPF patients was found to be 0.21 ± 0.08, which was significantly lower than the Jac-mean of healthy controls (0.27 ± 0.07, *p* < 0.001).

### Lung deformation and the severity of IPF

**Fig. 5:**
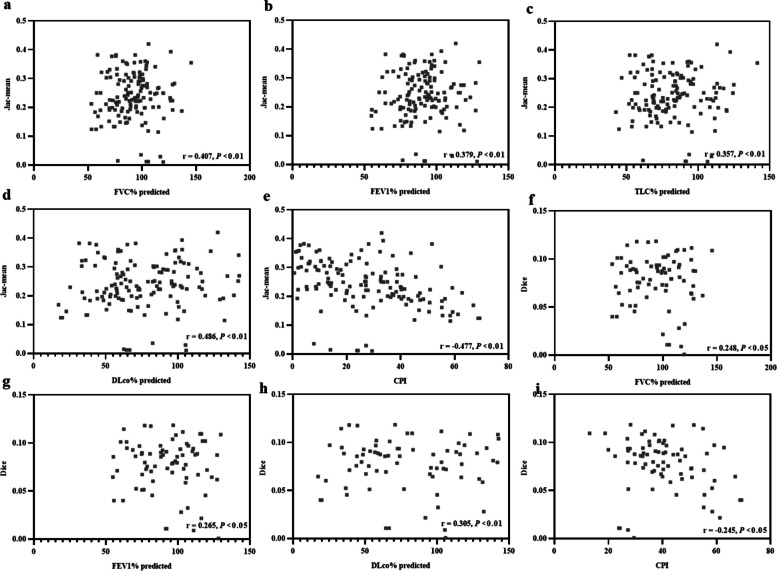
Relationship between the value of elastic registration (Jac-mean and Dice similarity coefficient) and measurements from pulmonary function tests. FVC, forced vital capacity; FEV1, forced expiratory volume; TLC, total lung capacity; DLCO, diffusing capacity of the lungs for carbon monoxide; CPI, composite physiological index

Figure 5 (a–e) indicated that the Jac-mean positively correlated with the FVC% predicted (*r* = 0.407, *p* < 0.01), FEV1% predicted (*r* = 0.379, *p* < 0.01), TLC% predicted (*r* = 0.357, *p* < 0.01), DLco% predicted (*r* = 0.486, *p* < 0.01), and negatively correlated with CPI (*r* = − 0.477, *p* < 0.01). Meanwhile, the Dice positively correlated with FVC% predicted (*r* = 0.248, *p* < 0.05), FEV1% predicted (*r* = 0.265, *p* < 0.05), DLco% predicted (*r* = 0.305, *p* < 0.05), and negatively correlated with CPI (*r* = − 0.245, *p* < 0.05) (Fig. 5f–i).

As shown in Table 2, the lung deformation in IPF patients with MRC ≥ 3 (Jac-mean: 0.16 ± 0.03; Dice: 0.06 ± 0.02) was significantly lower than MRC 1 (Jac-mean: 0.25 ± 0.03, *p* < 0.001; Dice: 0.10 ± 0.01, *p* < 0.001) and MRC 2 (Jac-mean: 0.22 ± 0.11, *p* = 0.001; Dice: 0.08 ± 0.03, *p* = 0.006). Meanwhile, the Jac-mean in patients with MRC 1 was higher than MRC 2 (*p* = 0.026); however, the Dice was comparable between MRC 1 and MRC 2 (*p* = 0.236).

**Table 2:** The characteristic of lung shrinkage is based on elastic registration between dyspnea score groups in patients with IPF

	Dyspnea score		
	MRC 1 (*n* = 24)	MRC 2 (*n* = 34)	MRC ≥ 3 (*n* = 18)
Jac-mean	0.25 ± 0.03	0.22 ± 0.11^a*^	0.16 ± 0.03^b*c*^
Dice	0.10 ± 0.01	0.08 ± 0.03^a^	0.06 ± 0.02^b*c*^

*IPF*, idiopathic pulmonary fibrosis; *MRC*, Medical Research Council

^a^The difference between MRC 1 and MRC 2

^b^The difference between MRC 2 and MRC ≥ 3

^c^The difference between MRC 1 and MRC ≥ 3

^*^p < 0.05

**Fig. 6:**
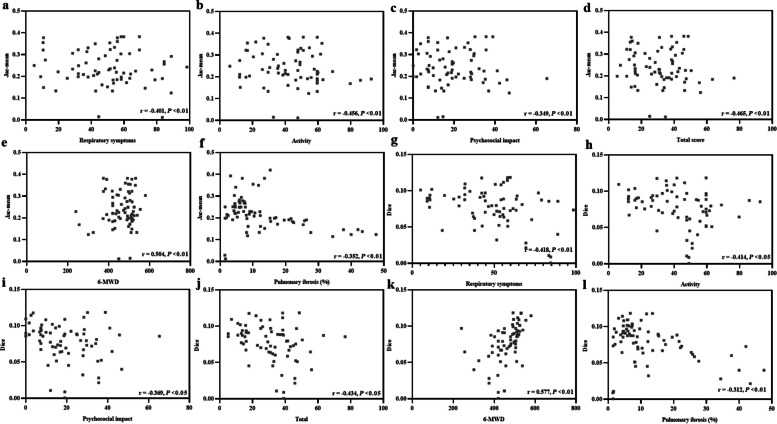
Relationship between Jac-mean (**a**–**f**), Dice similarity coefficient (**g**–**l**), and indicators of health-related quality of life, 6 min-walk distance (6-MWD), and the extent of pulmonary fibrosis on HRCT

In the aspect of health-related quality of life, the Jac-mean (Fig. 6a–d) was negatively correlated with respiratory symptoms (*r* = − 0.401, *p* < 0.01), activity (*r* = − 0.456, *p* < 0.01), psychosocial impact (*r* = − 0.349, *p* < 0.01), and total score (*r* = − 0.465, *p* < 0.01). Moreover, the Jac-mean also correlated with 6MWD (*r* = 0.504, *p* < 0.01) (Fig. 6e) and the extent of pulmonary fibrosis on HRCT(*r* = − 0. 352, *p* < 0.01 ) (Fig. 6f). The Dice similarity coefficient negatively correlated with respiratory symptoms (*r* = − 0.418, *p* < 0. 01), activity (*r* = − 0.414, *p* < 0.05), psychosocial impact (*r* = − 0.369, *p* < 0.05), and the total score (*r* = − 0.434,* p* < 0.05) (Fig. 6g–j) and also correlated with 6MWD (*r* = 0.577, *p* < 0.01) (Fig. 6k). The Dice similarity coefficient also negatively correlated with pulmonary fibrosis on HRCT (*r* = − 0. 312, *p* < 0. 01) (Fig. 6l).

As Table 3 shows, the Jac-mean correlated with the volume, number, and tortuosity of TPV (volume: *r* = 0.245, *p* < 0.01; number: *r* = 0.277, *p* < 0.01; tortuosity: *r* = − 0.320, *p* < 0.01), PVV (number: *r* = 0.188, *p* < 0.05; tortuosity: *r* = − 0.210, *p* < 0.05), and PAV (volume: *r* = 0.267, *p* < 0.01; number: *r* = 0.303, *p* < 0.01; tortuosity: *r* = − 0.350, *p* < 0.01), while the Dice similarity coefficient showed no correlation with pulmonary vascular-related indexes on HRCT.

**Table 3:** Relationship between the value of elastic registration (Jac-mean and Dice similarity coefficient) and pulmonary vascular-related indexes on HRCT

	Jac-mean		Dice	
	*r*	*p*	*r*	*p*
Total pulmonary vascular				
Volume (ml)	0.245	< 0.01^*^	− 0.146	> 0.05
Number	0.277	< 0.01^*^	− 0.168	> 0.05
Tortuosity	− 0.320	< 0.01^*^	− 0.050	> 0.05
Pulmonary vein vascular				
Volume (ml)	0.162	> 0.05	− 0.088	> 0.05
Number	0.188	< 0.05^*^	− 0.212	> 0.05
Tortuosity	− 0.210	< 0.05^*^	0.001	> 0.05
Pulmonary artery vascular				
Volume (ml)	0.267	< 0.01^*^	− 0.146	> 0.05
Number	0.303	< 0.01^*^	− 0.113	> 0.05
Tortuosity	− 0.350	< 0.01^*^	− 0.107	> 0.05

^*^p < 0.01 and ^*^p < 0.05 indicated have significance

## Discussion

In this study, we evaluated the lung deformation in IPF patients by elastic registration algorithm on 3D-UTE MRI and there are several findings: (I) the lung deformation decreased in the peripheral region of the lung bases in IPF patients; (II) the decreased lung deformation in IPF patients correlated with the worsen FVC%, FEV1%, TLC%, DLco%, and CPI; and (III) the decreased lung deformation in IPF patients correlated with the deteriorating 6-MWD, HRQoL, and the extent of lung fibrosis on HRCT.

During the progressive of interstitial fibrosis, normal interstitial tissue is replaced by an altered extracellular matrix and alveolar architecture, resulting in deterioration of lung compliance and decrease of lung deformation and elasticity [29, 30]. The elastic registration algorithm utilizes a dynamic linear elastic model to capture tissue deformation, which is discretized using the finite element method. In the context of follow-up for fibrotic interstitial lung disease, lung shrinkage has been employed as an additional CT marker [31]. In current study, the Jacobian maps from elastic registration showed the marked deformation areas mainly distributed in the dorsal aspect of lung bases in healthy controls and significantly decreased in the peripheral region of the lung bases in IPF patients, which were consistent with the decreased lung deformation in patient with systemic sclerosis-related ILD [16]. The Jac-mean represents the deformation of lung. The negative value indicates lung shrinkage, and the positive value indicates lung stretch. Moreover, the smaller Jac-mean is, the weaker the lung deformation is. The Jac-mean of IPF patients significantly decreased, indicating that the lung deformation deteriorated in IPF patients. These were consistent with Chassagnon et al. [16], and they reported that the lesser lung deformation of SSc patients compared with participants without fibrosis.

In order to accurately evaluate the value of lung deformation in IPF, we included functional and morphological parameters including PFT, CPI, Medical Research Council scale, HRQoL, 6-MWD, and the extent of fibrosis on HRCT. PFT is vital marker for evaluating the functional severity of IPF; however, it limits to provide the subtle and regional functional alteration as well as the extent of fibrosis. The increased value of Jac-mean and Dice similarity coefficient correlated with the increased FVC%, FEV1%, TLC%, and DLco%. Furthermore, the decreased value of Jac-mean and Dice similarity coefficient correlated with the increased CPI. These results demonstrated that decreased lung deformation is consistent with the deteriorated lung function. The Dice similarity coefficient is used to compare the similarity of the marked shrinkage area between IPF and healthy controls. Therefore, both Jac-mean (including color map) and Dice similarity coefficient can provide visual and quantitative analysis of regional or global lung function.

With the progress of IPF, the clinical symptoms and signs of the patient gradually deteriorate. We found the value of Jac-mean and Dice similarity coefficient decreased in patients with MRC3 and 4, compared with patients with MRC1 and 2. This revealed that the more lung deformation of IPF patients decreases, the more severe dyspnea they will have. Furthermore, we also found the value of Jac-mean and Dice similarity coefficient correlated with 6MWD, respiratory symptoms, activity, psychosocial impact, and the total score of IPF patients, confirming that decreased lung deformation correlated with worse exercise tolerance and the poor quality of life. In addition, Jac-mean had a weak correlation with the extent of pulmonary fibrosis and pulmonary vascular-related indexes on HRCT. These results confirmed that lung deformation based on elastic registration of UTE MRI correlated with clinical severity of IPF patients and further proved that the elastic registration on UTE MRI can evaluate morphological and functional alterations during the follow-up.

Accuracy and reproducibility of elastic registration are critical for the analysis of lung deformation in IPF patients. Chassagnon et al. used the distances between landmarks to evaluate the registration performance, validating the accuracy of the elastic registration [16]. In our study, we used the landmark distance and IoU to evaluate the accuracy of the elastic registration, where our resulting landmark distance is good as Chassagnon et al., and our IoU reaches 0.88 [16]. Furthermore, we go beyond the limitation of Chassagnon et al. [16] and verify that the elastic registration results are reproducible by acquiring images of two respiratory phases for each patient.

In this prospective study, we employed elastic registration of inspiratory-to-expiratory 3D-UTE MRI to assess the severity of IPF. However, it is important to acknowledge several limitations in our study. First, this study may restrict generalizability of results due to its single-center design and focus on predominantly mild cohort of patients. For patients with advanced or acute exacerbation of IPF, the lung deformation with prognosis needs further research. In our patients, we had no follow-up MR and the application of elastic registration in assessing the status of IPF (stable or progressive) need further research. Second, considering that the registration method we used is derived from open-source software (ANTs), it is sufficiently general. At the same time, we compared the registration accuracy of each participant’s two breathing phases to confirm that this registration method is reproducible. Nonetheless, the possible impact of different registration methods remains unknown.

In conclusion, lung deformation decreased in patients with IPF and correlated with the severity of IPF. Elastic registration of inspiratory-to-expiratory 3D UTE MRI may be a new morphological and functional marker for non-radiation and noninvasive evaluation of IPF.

### Supplementary Information

**Additional file 1:** **Supplementary figure 1.** The segmentation model for tissue characterization of idiopathic pulmonary fibrosis on HRCT. Ground-glass opacities (red), reticulation (orange), and honeycombing(green). **Supplementary figure 2.** The segmentation model for pulmonary vascular of healthy controls(a-d) and idiopathic pulmonary fibrosis(e-h) on HRCT. Total lung segmentation (a, e); pulmonary vascular (b, f); pulmonary vein(blue) and artery(red) vascular (c, g); the skeleton of pulmonary vein(blue) and artery(red) vascular (d, h). **Supplementary figure 3.** The picture showed the position of points A and B. The location of points A and B were manually drawn and derived from the anterior edge of the thoracic vertebra intersects the chest walls on both sides at the planer of the lower margin of the manubrium sterni on multiplanar reconstructions (MPR).

## Data Availability

The data used or analyzed during the current study are available from the corresponding author on reasonable request.

## Abbreviations

6-MWD: 6 min-walk distance
ALAT: Latin American Thoracic Association
ALI: Acute lung injury
ATS: American Thoracic Society
BMI: Body mass index
COPD: Chronic obstructive pulmonary disease
CPI: Composite physiological index
DLCO: Diffusing capacity of the lungs for carbon monoxide
ERS: European respiratory society
FEV1: Forced expiratory volume
FVC: Forced vital capacity
GGO: Ground-glass opacity
HRCT: High-resolution computed tomography
HRQoL: Health-related quality of life
ICC: Intraclass coefficients
IoU: Intersection over union
IPF: Idiopathic pulmonary fibrosis
JRS: Japanese Respiratory Society
MRC: Medical Research Council
MRI: Magnetic resonance imaging
PFTs: Pulmonary function tests
ROI: Regions of interest
SSc-ILD: Interstitial lung disease in patients with systemic sclerosis
UIP: Usual interstitial pneumonia
UTE: Ultrashort echo time
ZTE: Zero echo time
