# Supplementary material for: Assessment of lung deformation in patients with idiopathic pulmonary fibrosis with elastic registration technique on pulmonary three-dimensional ultrashort echo time MRI
Source: Insights Imaging. 2024 Jan 23;15:17. doi: 10.1186/s13244-023-01555-x (PMC10803694; doi:10.1186/s13244-023-01555-x)
Supplement: Supplementary file 1 — Additional file 1: Supplementary figure 1. The segmentation model for tissue characterization of idiopathic pulmonary fibrosis on HRCT. Ground-glass opacities (red), reticulation (orange), and honeycombing(green). Supplementary figure 2. The segmentation model for pulmonary vascular of healthy controls(a-d) and idiopathic pulmonary fibrosis(e-h) on HRCT. Total lung segmentation (a, e); pulmonary vascular (b, f); pulmonary vein(blue) and artery(red) vascular (c, g); the skeleton of pulmonary vein(blue) and artery(red) vascular (d, h). Supplementary figure 3. The picture showed the position of points A and B. The location of points A and B were manually drawn and derived from the anterior edge of the thoracic vertebra intersects the chest walls on both sides at the planer of the lower margin of the manubrium sterni on multiplanar reconstructions (MPR). [file 13244_2023_1555_MOESM1_ESM.docx]

**Assessment of lung deformation in patients with idiopathic pulmonary fibrosis with elastic registration technique on pulmonary three-dimensional ultrashort echo time MRI**

**ELECTRONIC SUPPLEMENTARY MATERIAL**


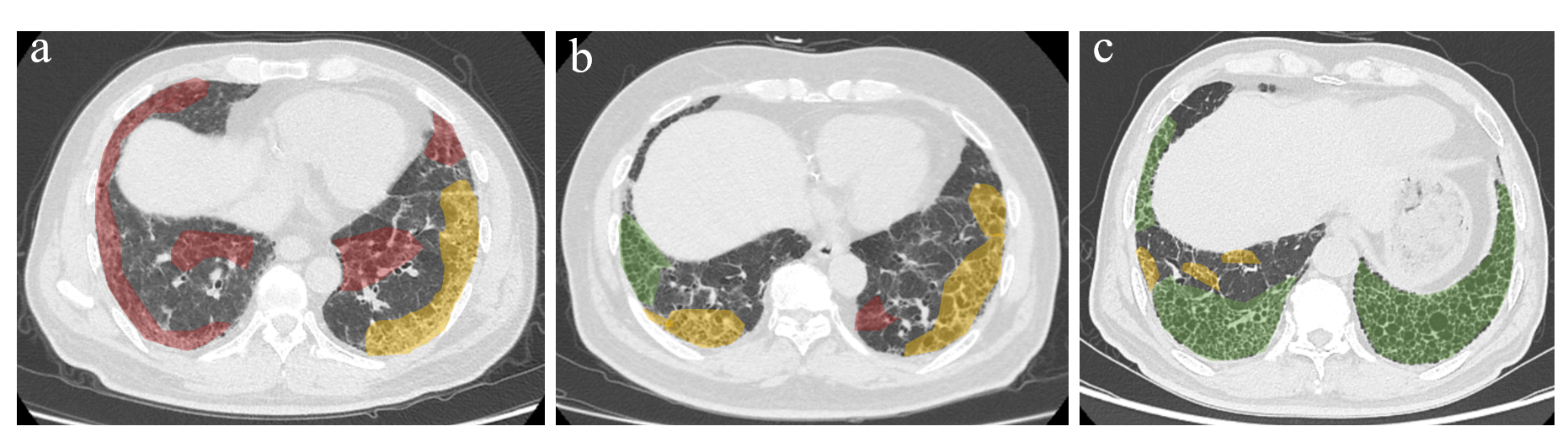


**Supplementary figure 1**. The segmentation model for tissue characterization of idiopathic pulmonary fibrosis on HRCT. Ground-glass opacities (red), reticulation (orange), and honeycombing(green).


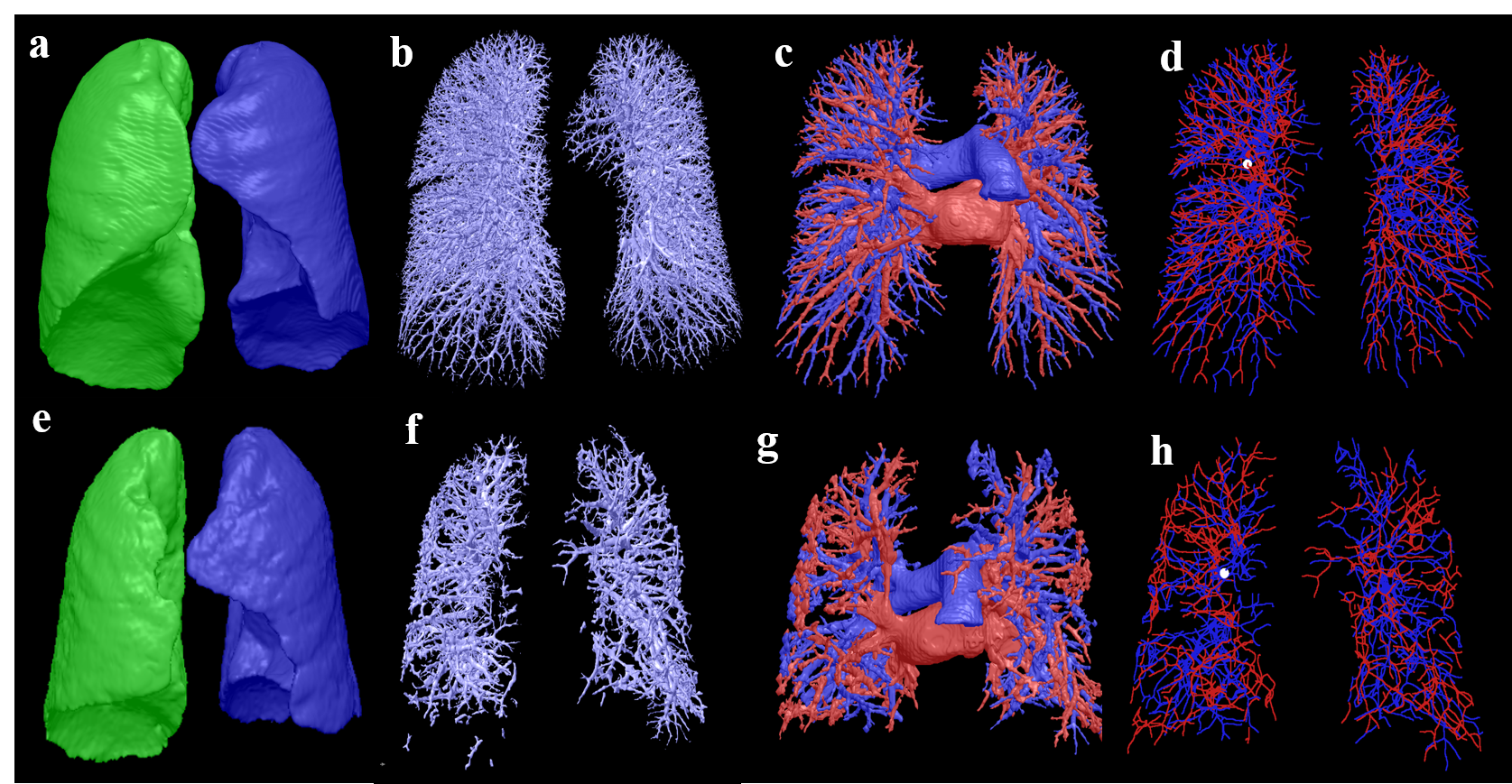


**Supplementary figure 2**. The segmentation model for pulmonary vascular of healthy controls(a-d) and idiopathic pulmonary fibrosis(e-h) on HRCT. Total lung segmentation (a, e); pulmonary vascular (b, f); pulmonary vein(blue) and artery(red) vascular (c, g); the skeleton of pulmonary vein(blue) and artery(red) vascular (d, h).


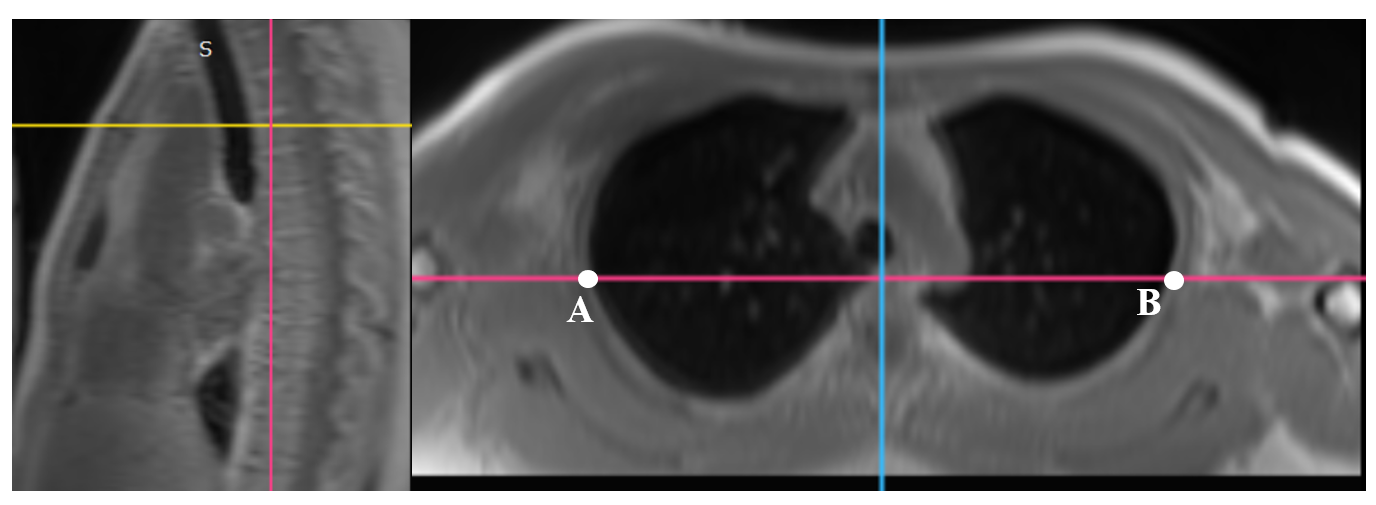


**Supplementary figure 3**. The picture showed the position of points A and B. The location of points A and B were manually drawn and derived from the anterior edge of the thoracic vertebra intersects the chest walls on both sides at the planer of the lower margin of the [manubrium sterni](javascript:;) on multiplanar reconstructions (MPR).
